# Predicting the Severity of Symptoms of the COVID Stress Syndrome From Personality Traits: A Prospective Network Analysis

**DOI:** 10.3389/fpsyg.2021.632227

**Published:** 2021-04-29

**Authors:** Steven Taylor, Allan Fong, Gordon J. G. Asmundson

**Affiliations:** ^1^Department of Psychiatry, University of British Columbia, Vancouver, BC, Canada; ^2^Department of Psychology, University of Regina, Regina, SK, Canada

**Keywords:** COVID-19, COVID Stress Syndrome, personality, intolerance of uncertainty, health anxiety, resilience, negative emotionality, network analysis

## Abstract

Psychological stress reactions to the COVID-19 pandemic are complex and multifaceted. Research provides evidence of a COVID Stress Syndrome (CSS), consisting of (1) worry about the dangerousness of getting infected with SARSCoV2 and coming into contact with infected surfaces, (2) worry concerning the personal socioeconomic consequences of COVID-19, (3) xenophobic fears that SARSCOV2 is being spread by foreigners, (4) COVID-19-related traumatic stress symptoms (e.g., nightmares), and (5) compulsive checking and reassurance-seeking about COVID-19. Little is known about how these symptoms are related to vulnerability and protective personality factors. Based on data from 1,976 US and Canadian adults, we conducted a prospective network analysis in which personality factors were initially assessed at Time 1 and then symptoms of the CSS were assessed at Time 2, 2.5 months later. Results indicated that trait optimism and trait resilience were negatively associated with negative emotionality, suggesting a modulatory (inhibitory) influence. Negative emotionality was positively linked to the narrower traits of intolerance of uncertainty and health anxiety proneness. These narrower traits, in turn, were prospectively linked to symptoms of the CSS. Results suggest that the effects of broad personality traits (e.g., negative emotionality, trait resilience) on symptoms of the CSS were mediated by narrower traits such as the intolerance of uncertainty. Treatment implications are discussed.

## Highlights

- Results support the concept of the COVID Stress Syndrome (CSS).
- Conducted a prospective network analysis of trait predictors of CSS.
- Trait optimism and resilience modulated the effects of negative emotionality on CSS.
- Negative emotionality was linked indirectly to the CSS via narrower traits.
- Intolerance of uncertainty and health anxiety proneness were directly linked to CSS.

## Introduction

The understanding of COVID-19-related distress has rapidly evolved since the outbreak of the novel coronavirus. Initially, COVID-19-related distress was conceptualized narrowly, as a form of specific phobia (“coronaphobia”) or a similarly narrowly defined anxiety-related phenomenon, whereas later research has shown that COVID-19-related distress is far more complex and multifaceted (Asmundson and Taylor, 2020). A growing body of research provides evidence of what has been called a COVID Stress Syndrome (CSS), which does not neatly fit into existing DSM-5 diagnostic categories (Taylor et al., 2020a,b). The syndrome is essentially dimensional in terms of severity (Taylor et al., 2020a), although for diagnostic purposes people can be classified as having a COVID Stress Disorder if they have severe impairment in social or occupational functioning due to COVID-19-related distress (Asmundson and Taylor, 2020). It is currently unclear whether this disorder is a form of adjustment reaction that abates when the COVID-19 pandemic subsides, or whether it will become chronic for some people. The CSS is currently conceptualized as an adjustment disorder, but that does not imply that it is evanescent, because some adjustment disorders can transform into chronic conditions (Taylor, 2021).

Given that the CSS is essentially dimensional in nature, researchers have investigated it in terms of severity (Taylor et al., 2020a). The syndrome consists of five intercorrelated elements, as assessed by the five COVID Stress Scales: (1) Worry concerning the dangerousness of COVID-19 along with worry about coming into contact with fomites (i.e., objects, surfaces) potentially contaminated with SARSCoV2, (2) worry concerning the personal socioeconomic consequences of the COVID-19 pandemic (e.g., worry about disruption in the supply chain, worry about personal finances), (3) xenophobic fears that SARSCOV2 is being spread by foreigners, (4) traumatic stress symptoms associated with vicarious or direct traumatic exposure to COVID-19 (i.e., COVID-19-related nightmares, intrusive thoughts or images), and (5) COVID-19-related reassurance-seeking and compulsive checking (Taylor et al., 2020a,b).

Research suggests that the severity of the CSS is associated with premorbid (i.e., pre-COVID-19 pandemic) mental health problems (Asmundson et al., 2020), although much remains to be learned about the links between these problems and specific symptoms of the CSS. Similarly, much remains to be learned about the relationship between personality traits and the CSS. Personality traits can be vulnerability factors for psychopathology or protective, stress-buffering factors that enable the person to cope with life stressors without developing psychopathology. Trait optimism and trait resilience are buffering factors against stressors in general (Connor and Davidson, 2003; Coelho et al., 2018). The most well-established vulnerability factor is negative emotionality (neuroticism), which is a broad trait conferring vulnerability for all kinds of psychopathology (Brandes et al., 2019). Although negative emotionality is composed of facets (narrow traits), research supports of bifactor model of negative emotionality, consisting of a general factor in addition to distinct, but correlated, narrow factors (Subica et al., 2016; Brandes et al., 2019; Fournier et al., 2019).

Proneness to health anxiety and intolerance of uncertainty are narrow factors, correlated with, but conceptually and empirically distinguishable from negative emotionality (Taylor and Asmundson, 2004; Carleton et al., 2007; Taylor, 2019). Research from recent pandemics, including the COVID-19 pandemic, shows that negative emotionality, intolerance of uncertainty, and proneness to health anxiety are correlated with pandemic-related distress (Taylor, 2019; Lee and Crunk, 2020; Rettie and Daniels, 2020; Taylor et al., 2020a). Research further suggests that trait optimism and trait resilience may serve as buffers against the effects of pandemic-related distress (Taylor, 2019; Barzilay et al., 2020; Paredes et al., 2021). Little is known about how such traits are related to specific symptoms of the CSS.

Network analysis can provide insights into the interrelationships among variables. In fact, a network approach makes theoretical sense in terms of cognitive-behavioral models of health anxiety, pandemics, and trauma-related fears (Taylor and Asmundson, 2004; Taylor, 2017, 2019). This is because these models predict that nodes in the network interact with one another. For example, negative beliefs or expectations (e.g., worry about COVID-19 infection and its sources and consequences) give rise to COVID-19-related checking for information about the seriousness of the threat and how best to cope. Checking, in turn, can exacerbate worries about the threat of COVID-19, because checking (e.g., checking for health-related information online) inevitably backfires, leading the person to encounter new, fear-evoking information (e.g., images or descriptions of sickness and death in the mainstream news or social media), which in turn amplify worries (Taylor, 2019; Taylor et al., 2020a). Exposure to graphic news stories can also give rise to traumatic stress symptoms, such as nightmares and intrusive thoughts and images. Reexperiencing symptoms, in turn, can increase the perceived threat, because reexperiencing provides vivid reminders of the dangerousness of COVID-19. The propensity to experience symptoms of the CSS is likely to be influenced by various personality traits, as discussed above, although the nature of the interrelationships remains to be elucidated.

Given these considerations, the present study examined how the above-mentioned personality traits (negative emotionality, trait optimism, trait resilience, intolerance of uncertainty, and proneness to health anxiety) are related specifically to symptoms of the CSS. Although other traits are potentially relevant to understanding COVID-19-related distress, practical considerations (e.g., logistic constraints on the size of the assessment battery), precluded the evaluation of other traits. However, we also examined the effects of past history of general medical conditions and mental health condition on the symptoms of the CSS.

A novel aspect of the present study is that the relationships between personality traits and symptoms of the CSS were investigated by conducting a prospective network analysis, where trait vulnerability and protective factors were assessed at Time 1 and symptoms of the CSS were assessed later, at Time 2. Network analysis yields important information about relationships among its elements (e.g., relationships among personality traits and symptoms), assuming that nodes (e.g., symptoms, traits, or other variables) cluster together because they are somehow causally related to one another. The links between nodes are called “edges.” Network analysis does not assume that nodes are influenced by some underlying factor such as a latent variable. Instead, network analysis assumes that nodes can influence one another via their edges (Epskamp et al., 2018). If nodes causally influence one another, then changes in a central node will lead to changes in other nodes through a spreading of activation throughout the network. Central nodes are defining features of a network; as such, identifying the most central nodes has the potential to inform which elements to target in interventions. As a caveat, it is important to note that, even with prospective designs such as the present study, results of network analyses suggest but do not establish causality. Significant edges could represent causal links but experimental designs are needed to establish causality. Therefore, network analyses provide a source of hypotheses about complex causalities among variables, which can then be examined in more detail using experimental designs.

## Materials and Methods

### Sample

The sample consisted of 1,976 adults from the United States (n = 988) and Canada (n = 988). The mean age was 54 years (*SD* = 14 years, range 18–99 years). Most (82%) had completed full or partial college, most (93%) were employed full- or part-time, and 40% were female. Most (70%) were Caucasian, with the remainder being African American/Black (8%), Asian (12%), Latino/Hispanic (6%), and other (4%). Only 2% of the sample reported being diagnosed with COVID-19. A total of 43% had a preexisting medical condition, 14% had a pre-existing (past year) mental health disorder, and 13% currently met criteria for COVID Stress Disorder.

### Data Collection Procedures

Data were collected at two timepoints (May 6–19 and July 20-August7, 2020), separated by a mean of 2.5 months, using an internet-based self-report survey delivered in English by Qualtrics, which is a commercial survey sampling and administration company. All participants completed assessments at both timepoints. Qualtrics solicited this adult sample as part of our research program concerning the psychology of COVID-19 (Taylor et al., 2020a,b). Qualtrics maintains a pool of survey participants and selects them to meet sampling quotas based on age, gender, ethnicity, socioeconomic status, and geographic region within each country. Items were used to identify and eliminate data from careless or incomplete responders. This included four items assessing whether participants were paying attention to the instructions (e.g., “This is an attention check, please select Strongly Agree”). To be included in the study, participants had to provide correct responses to three or more of the four attention check items. Also, at the end of the assessment battery participants were asked to indicate whether, in their honest opinion, we should use their data. Those who responded “no” were excluded from the study.

Incomplete item responses were rare (<5% per scale). Missing data were imputed via expectation-maximization. Respondents provided written informed consent prior to completing the survey. The Research Ethics Board of the University of Regina (REB# 2020-043) approved the research reported in this article.

### Measures

Participants completed demographic questions along with the measures included in the network analysis. Vulnerability factors (described below) were assessed at the first time point and symptoms of the CSS were assessed at the second time point. Scales measuring vulnerability factors were as follows: Negative emotionality was assessed by the Ten Item Personality Inventory (Gosling et al., 2003). The scale has performed well on various indices of reliability and validity (Gosling et al., 2003; Ehrhart et al., 2009; Nunes et al., 2018). Trait optimism was measured by the Optimism Scale (Coelho et al., 2018), which has been previously shown to have good reliability and validity (Coelho et al., 2018). Trait resilience was assessed by the Connor-Davidson Resilience Scale (Connor and Davidson, 2003), which has good psychometric properties (Connor and Davidson, 2020). The tendency to worry about one’s health in general (health anxiety proneness) was measured by the Short Health Anxiety Inventory, which has been shown to be psychometrically sound (Salkovskis et al., 2002). Intolerance of uncertainty was measured by the Intolerance of Uncertainty Scale-12, which also has good psychometric properties (Carleton et al., 2007). The presence (vs. absence) of a pre-existing general medical condition (e.g., heart disease) was assessed by a yes/no item, as was the presence (vs. absence) of a current (past-year) mental health condition. Symptoms of the CSS were assessed by the five COVID Stress Scales, as described earlier in this article, which have very good reliability and validity (Taylor et al., 2020b).

For each multi-item scale, ω total (McDonald, 1999) was used as the measure of reliability as internal consistency. McDonald’s ω was used instead of Cronbach’s α because the latter tends to underestimate reliability (McNeish, 2018). Values of ω are interpreted as follows: Values of 0.70–0.80 indicate acceptable reliability, 0.80–0.90 indicate good reliability, and values greater than 0.90 indicate excellent reliability. The obtained values of ω are presented along the diagonal of Table 1. Here it can be seen that the scales had excellent or good-to-excellent reliabilities.

**TABLE 1:** Correlations among variables (nodes) in the network analysis. Reliabilities (ω) for multi-item scales are in parentheses.

	MH	MED	RES	OPT	HA	IU	N	DAN	SEC	XEN	TSS	CHECK
MH	–											
MED	0.18***	–										
RES	−0.23***	0.00	–									
OPT	−0.26***	–0.04	0.74***	(0.94)								
HA	0.31***	0.20***	−0.33***	−0.36***	(0.92)							
IU	0.26***	0.08	−0.34***	−0.36***	0.51***	(0.93)						
N	0.37***	0.04	−0.61***	−0.58***	0.43***	0.43***	(0.88)					
DAN	0.11***	0.04	−0.14***	−0.14***	0.44***	0.39***	0.20***	(0.96)				
SEC	0.09***	0.04	−0.12***	−0.14***	0.36***	0.31***	0.16***	0.69***	(0.95)			
XEN	0.04	0.00	−0.08***	−0.09***	0.29***	0.28***	0.13***	0.58***	0.55***	(0.96)		
TSS	0.19***	–0.01	−0.15***	−0.17***	0.47***	0.41***	0.25***	0.57***	0.56***	0.43***	(0.96)	
CHECK	0.05	–0.06	–0.01	0.00	0.33***	0.27***	0.11***	0.49***	0.50***	0.40***	0.65***	(0.91)

*p < 0.01, **p < 0.005, ***p < 0.001.

*CHECK, COVID compulsive checking; DAN, Worry about the dangerousness of COVID; HA, Health anxiety proneness; IU, Intolerance of uncertainty; MED, Pre-existing medical condition; MH, Past year mental health condition; N, Negative emotionality; OPT, Trait optimism; RES, Trait resilience; SEC, Worry about socioeconomic impact; TSS, COVID traumatic stress symptoms; XEN, COVID xenophobia.*

### Statistical Analyses

Glasso networks, computed as networks of statistically significant (*p* < 0.01) edges (regularized partial correlations), were computed using the *qgraph* package in R (Epskamp et al., 2016). The “strength” index of centrality, also calculated with *qgraph*, was used to identify the most central nodes in the network. Although there are other indicators of centrality, strength has the most support as a stable and reliable indicator of centrality (Epskamp et al., 2018). For a given node, its strength was calculated by summing the absolute values of edges that connect that node with other nodes. A central node is one the highest strength value.

Node centrality difference tests, which determine whether some nodes in the network are significantly more central than other nodes, were calculated using the R *bootnet* package (Epskamp et al., 2016). To assess the stability (reliability) of the strength values for the nodes and their links, the Correlation of Stability coefficient was calculated via *bootnet* (Epskamp et al., 2018). Given the number of computations in this study (e.g., tests of statistical significance), the alpha level was set at 0.01.

**FIGURE 1:**
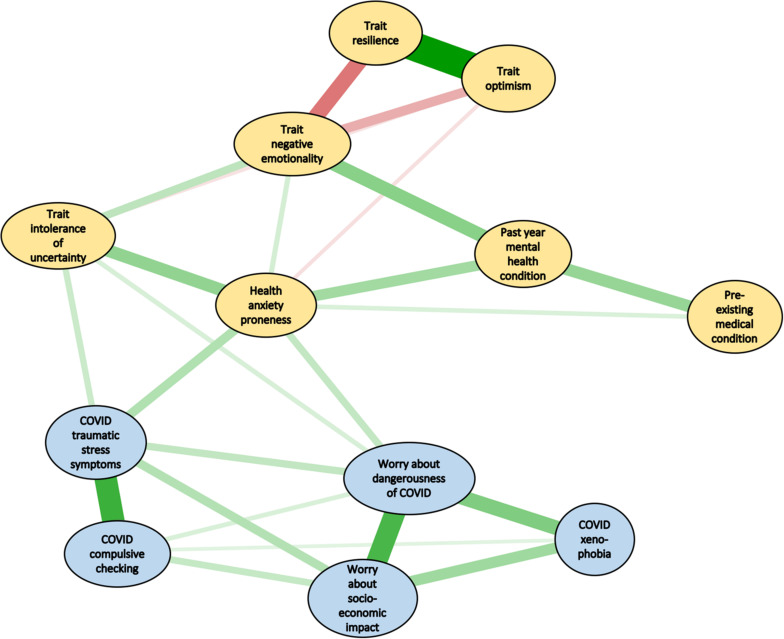
Network analysis of vulnerability variables (yellow ellipses) and symptoms of the COVID Stress Syndrome (CSS) (blue ellipses). The size and thickness of lines indicate the degree of strength of connections. Green lines indicate positive connections (i.e., positive regularized partial correlations), whereas red lines indicate negative connections.

With regard to the tuning parameters, which dictate network sparcity, the lambda min ratio was set at the default of 0.01 and the tuning parameter was set at the default of 1.0. Various ranges of these parameters were then explored, within conventional limits (Epskamp and Fried, 2018). The results did not appreciably change from those obtained with the default values, most likely because the network with default values provided a sparse network with theoretically meaningful edges (see Figure 1). Bootstrapping for the various analyses involved 2,500 bootstraps per test. Given this high bootstrapping value, the results did not change when an even higher bootstrapping value was used.

## Results

### Preliminary Analyses

For descriptive purposes, the correlations among variables in the network analysis are shown in Table 1. The table shows that most correlations were statistically significant and for more than a third (38%) their absolute values were medium-to-large in size (| *r*s| > 0.30), according to Cohen’s criteria (Cohen, 1988). All correlations among the five nodes of the CSS were positive and large (*r*s > 0.50), as would be expected from a syndrome of closely interrelated variables. The absolute values of the correlations among the trait predictors were medium-to-large.

### Network Analyses

Figure 1 shows the edges between nodes in the network (all *p*s < 0.01). The magnitude of the edges is indicated by shorter, thicker lines, with positive associations in green and negative ones in red. The numerical values of the edge and their significance levels appear in Table 2. The Correlation of Stability coefficients were 0.75 for both nodes and edges, which both exceed the cutoff of 0.50 (Epskamp et al., 2018), suggesting that the estimates of the relative magnitudes of nodes and edges were reliable. Note that because all of the edges in Figure 1 are regularized partial correlations, they represent a form of mediator analysis, controlling for the effects of other variables. So, for example, the edge connecting trait intolerance of uncertainty with health anxiety proneness (Figure 1) is a regularized partial correlation that controls for the effects of other nodes on those two variables. The purpose of network analysis is not to conduct a formal Baron-Kenny type of mediator analysis (Barron and Kenny, 1986), but nevertheless the network analysis efficiently reveals mediated effects, in which the links between two nodes simultaneously control for links among all other nodes.

**TABLE 2:** Edge weights (regularized partial correlations) between nodes in the network.

Edge	Weight
IU-HA	0.23***
IU-N	0.14***
IU-TSS	0.11***
IU-DAN	0.08***
IU-OPT	−0.06*
TSS-HA	0.16***
TSS-CHECK	0.41***
TSS-DAN	0.13***
TSS-SEC	0.16***
CHECK-DAN	0.08***
CHECK-XEN	0.06*
CHECK-SEC	0.12***
HA-N	0.09***
HA-MH	0.19***
HA-OPT	−0.07**
HA-DAN	0.13***
HA-MED	0.08***
OPT-RES	0.53***
N-RES	−0.29***
N-OPT	−0.17***
N-MH	0.25***
MH-MED	0.22***
SEC-DAN	0.38***
SEC-XEN	0.20***
DAN-XEN	0.27***

*p < 0.01, **p < 0.005, ***p < 0.001.

*CHECK, COVID compulsive checking; DAN, Worry about the dangerousness of COVID; HA, Health anxiety proneness; IU, Intolerance of uncertainty; MED, Pre-existing medical condition; MH, Past year mental health condition; N, Negative emotionality; OPT, Trait optimism; RES, Trait resilience; SEC, Worry about socioeconomic impact; TSS, COVID traumatic stress symptoms; XEN, COVID xenophobia.*

**FIGURE 2:**
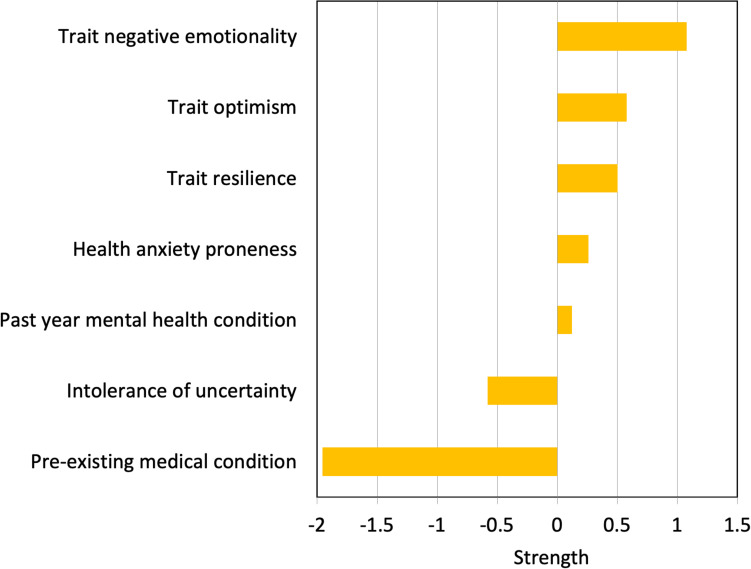
Strength of connection among nodes representing vulnerability factors.

**FIGURE 3:**
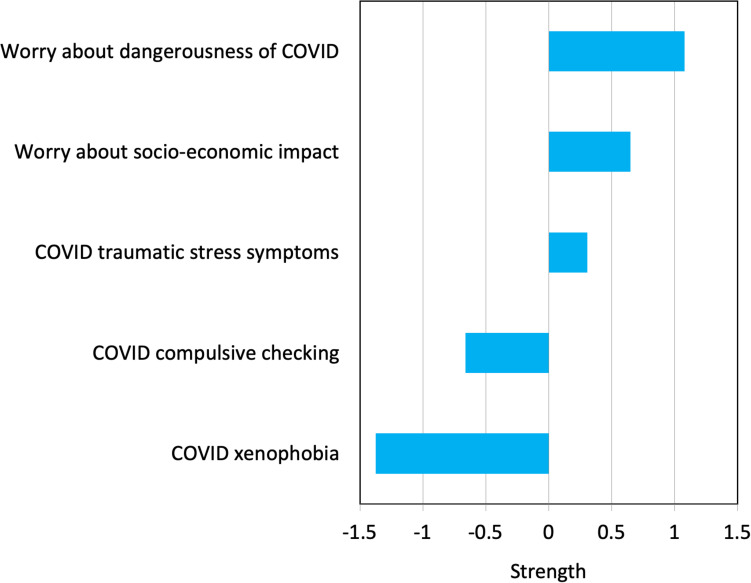
Strength of connection among nodes of the COVID Stress Syndrome (CSS).

Strength values for the sub-network of vulnerability factors (yellow ellipses in Figure 1) are shown in Figure 2. Here it can be seen that negative emotionality is central to that sub-network, as indicated by the largest value in Figure 2. The centrality indices (strength values) for the sub-network of COVID stress symptoms (blue ellipses in Figure 1) are shown in Figure 3. Here it can be seen that worry about the dangerousness of COVID-19 is central to that sub-network, as indicated by the largest value in Figure 3.

As shown in Figure 1, the results indicated two sub-networks, with negative emotionality at the center of the sub-network of vulnerability factors, and worry about the dangerousness of COVID-19 at the center of the sub-network of COVID stress symptoms. The links among variables in the network make conceptual sense. Trait resilience and trait optimism have strong positive associations with one another and both have negative (inhibitory) associations with negative emotionality and, to a lesser extent, negative associations with trait intolerance of uncertainty and health anxiety proneness. Not surprisingly, trait negative emotionality was linked to having a past-year mental health condition. Pre-existing mental health conditions and general medical conditions are also positively linked to health anxiety proneness.

The link between negative emotionality and the symptoms of the CSS was mediated thought health anxiety proneness and intolerance of uncertainty. That is, negative emotionality was not directly linked to symptoms of the CSS. Rather, it was linked indirectly though health anxiety and intolerance of uncertainty. The symptoms of the CSS were all strongly connected (i.e., significant edges; see also Table 1).

The most peripheral node in the network was the history of a preexisting medical condition (Figure 1), which also had the smallest and mostly non-significant correlations with other nodes (Table 2). This was an omnibus measure of past medical history, which was related, in theoretically expected ways, with past history of a mental health condition and with trait health anxiety proneness. Chronic diseases and other preexisting medical conditions are well-known contributors to poor mental health (e.g., depression) and health anxiety (Taylor and Asmundson, 2004).

## Discussion

Replicating previous research (Taylor et al., 2020a), we found that the nodes of CSS form a tightly connected network, at the center of which is worry about the dangerousness of COVID-19. The center of the trait network was negative emotionality. Results of the prospective network analysis further indicated that trait optimism and trait resilience were negatively associated with negative emotionality, suggesting a modulatory influence. Negative emotionality was positively linked to the narrower traits of intolerance of uncertainty and health anxiety proneness. These narrower traits, in turn, were prospectively linked to symptoms of the CSS. Results suggest that the effects of broad personality traits (e.g., negative emotionality, trait resilience) on symptoms of the CSS were mediated by narrower traits such as the intolerance of uncertainty.

Findings from this study are consistent with theory and research about health anxiety in general (Taylor and Asmundson, 2004); specifically, that proneness to health anxiety is influenced by negative emotionality. In the present study, trait negative emotionality measured in May 2020 directly and indirectly (through intolerance of uncertainty and past year mental health conditions) influenced health anxiety which, in turn, impacted the severity of CSS in August 2020. The current findings are also consistent with research on pandemic-related fear in earlier (pre-COVID-19) pandemics, where it was found that the personality traits investigated in the present study were related to pandemic-related fear (Taylor, 2019). The present study builds on previous research by identifying a patterned network of inter-relations, where some traits are directly linked to the CSS while other traits are indirectly linked to the syndrome.

If the connections among nodes are causally related, then the findings suggest reducing intolerance of uncertainty and health anxiety proneness may have downstream beneficial effects in reducing symptoms of the CSS. However, the results of the network analysis suggest that a more efficient means of reducing symptoms of CSS (and COVID Stress Disorder) would be to target general vulnerability factors; that is, building optimism and resilience and reducing negative emotionality, which (if the network links are causal in nature) would reduce COVID-related stress symptoms as well as the intolerance of uncertainty and health anxiety proneness. This could be accomplished in a number of ways, such as by using transdiagnostic cognitive-behavior therapy to target negative emotionality and other vulnerability traits, as well as cognitive-behavioral and other methods for building resilience and optimism (Segerstrom, 2007; Zoellner and Feeny, 2014; Barlow and Farchione, 2017).

The present study has strengths and limitations. Regarding the strengths, the sample was large and the present study appears to be the first to use prospective network analysis to understand the interrelationships among vulnerability and protective traits and the symptoms of the CSS. The links found in this study made conceptual sense and are consistent with cognitive-behavioral approaches for understanding health anxiety, traumatic stress symptoms, and pandemic-related behaviors (Taylor and Asmundson, 2004; Taylor, 2017, 2019). A limitation is that not all potentially relevant traits were assessed. Potentially relevant traits for understanding pandemic-related stress include the traits of harm avoidance, overestimation of threat, and perfectionism (Taylor, 2019). Further research is needed to investigate their potential links to the symptoms of the CSS. The replicability of the findings across different countries and cultures also remains to be investigated in future research.

Additional research is needed to determine whether the findings of the present study, of which only 2% of participants were diagnosed with COVID-19, generalize to samples consisting entirely of patients diagnosed with COVID-19. Research suggests that infection with SARSCoV2 is associated with a heightened risk of psychopathology (Taquet et al., 2020). It is currently unclear whether personality traits such as those investigated in the present study play a role of exacerbating or buffering COVID-19-induced psychopathology. Variations as a function of demographics also remain to be investigated. Our sample, with a mean age of 54 years is representative of the age of adults in the US and Canada, according to census data of adults (>17 years) (e.g., https://www.census.gov/data/tables/time-series/demo/popest/2010s-national-detail.html). Nevertheless, the question arises as to whether the pattern of results vary across age groups and other demographic groups.

Finally, prospective network analysis, as a statistical modeling approach, is not sufficient for determining the causal status of nodes. Nevertheless, the present findings provide a strong rationale for conducting future experimental studies on the causal status of vulnerability and protective traits in shaping the severity of symptoms of the CSS.

## Data Availability Statement

The raw data supporting the conclusions of this article will be made available by the authors, without undue reservation.

## Ethics Statement

The studies involving human participants were reviewed and approved by the Research Ethics Board of the University of Regina. The patients/participants provided their written informed consent to participate in this study.

## Author Contributions

ST: conceptualization, formal analysis, funding acquisition, supervision, methodology, writing—original draft, and writing—review and editing. AF: writing—original draft and writing—review and editing. GA: funding acquisition, methodology, data curation, supervision, writing—original draft, and writing—review and editing. All authors contributed to the article and approved the submitted version.

## Conflict of Interest

The authors declare that the research was conducted in the absence of any commercial or financial relationships that could be construed as a potential conflict of interest.
